# Development of priorities for a Canadian strategy to advance activity-based therapies after spinal cord injury

**DOI:** 10.1038/s41393-021-00644-2

**Published:** 2021-06-07

**Authors:** Kristin E. Musselman, Kristen Walden, Vanessa K. Noonan, Hope Jervis-Rademeyer, Nancy Thorogood, Laurent Bouyer, Brian Chan, Sarah Donkers, Chester Ho, Tara Jeji, Anita Kaiser, Tara D. Klassen, José Zariffa, Christopher Grant, Kei Masani, Dominik Zbogar, Peter Athanasopoulous, and the Canadian ABT Summit Attendees

**Affiliations:** 1 Department of Physical Therapy, University of Toronto, Toronto, ON Canada; 2 KITE, Toronto Rehabilitation Institute-University Health Network, Toronto, ON Canada; 3 Rehabilitation Sciences Institute, University of Toronto, Toronto, ON Canada; 4 Praxis Spinal Cord Institute, Vancouver, BC Canada; 5 Department of Rehabilitation, Laval University, Quebec City, QC Canada; 6 Center for Interdisciplinary Research in Rehabilitation and Social Integration, Quebec City, QC Canada; 7 Institute of Health Policy, Management and Evaluation, University of Toronto, Toronto, ON Canada; 8 School of Rehabilitation Science, University of Saskatchewan, Saskatoon, SK Canada; 9 Department of Medicine, University of Alberta, Edmonton, AB Canada; 10 Physical Medicine and Rehabilitation, Glenrose Rehabilitation Hospital, Edmonton, AB Canada; 11 Ontario Neurotrauma Foundation, Toronto, ON Canada; 12 Canadian Spinal Research Organization, Toronto, ON Canada; 13 Department of Physical Therapy, University of British Columbia, Vancouver, BC Canada; 14 GF Strong Rehabilitation Centre, Vancouver, BC Canada; 15 Institute of Biomedical Engineering, University of Toronto, Toronto, ON Canada; 16 Cumming School of Medicine, University of Calgary, Calgary, AB Canada; 17 Foothills Medical Centre, Calgary, AB Canada; 18 Spinal Cord Injury Rehabilitation Evidence, Vancouver, BC Canada; 19 Spinal Cord Injury Ontario, Toronto, ON Canada; 20 SCI Solutions Alliance, Toronto, ON Canada

**Keywords:** Rehabilitation, Spinal cord diseases

## Abstract

**Study Design:**

Participatory design.

**Objectives:**

Activity-based therapies (ABT) have physical and psychosocial benefits for individuals with spinal cord injury (SCI). A Canadian ABT summit was held to: (1) identify methods used in stroke rehabilitation that may be appropriate for SCI; (2) understand the current state of ABT activities in Canada; and (3) identify priorities for ABT research and care for the next five years.

**Setting:**

Stakeholder-engaged meeting at a tertiary rehabilitation hospital.

**Methods:**

Thirty-nine stakeholders, including individuals with SCI, frontline clinicians, healthcare administrators, researchers, funders and health policy experts, attended. Two participants were note-takers. Priority identification occurred through input from stakeholder groups, followed by individual voting. Conventional content analysis was used to synthesize the information in the meeting notes.

**Results:**

The strengths of ABT in stroke rehabilitation included clear and clinically feasible definitions, measurements and interventions, and recognized requirements for implementation (e.g. behavior change, partnerships). Knowledge gaps concerning ABT activities in Canada were identified for acute and community settings, non-traumatic populations, and the interventions, equipment and standardized measures (i.e. upper limb, activity levels) used. Five priorities for ABT across the continuum of care were identified: (1) Identify current ABT activities; (2) Create a network to facilitate dialog; (3) Track engagement in ABT activities; (4) Develop and implement best practice recommendations; and (5) Study optimal timing, methods, and dose of ABT. Working groups were formed to address priorities 1–3.

**Conclusions:**

The priorities will guide SCI research and care activities in Canada over the next five years.

**Sponsorship:**

Praxis Spinal Cord Institute.

## Introduction

Spinal cord injury (SCI) is a life-altering condition associated with low prevalence and high cost. Roughly 86,000 Canadians live with the effects of SCI [1], which often include significant impairments in motor, sensory and autonomic functions. Until recently, the neurological damage and resulting loss of function associated with SCI were believed to be permanent. Consequently, rehabilitation following SCI was focused on the resumption of daily activities through compensatory mechanisms. More recently, however, the focus of SCI rehabilitation has shifted from compensation to recovery, whereby rehabilitation interventions aim to target areas of the body affected by the injury with the intent of regaining function through use of the affected limbs and trunk. This shift in the rehabilitative approach resulted from an increased understanding of the potential for neuroplasticity following SCI. Neuroplasticity is the ability of the nervous system to change, structurally and/or functionally, in response to a variety of triggers, such as injury, pharmacological stimulation, movement, and exercise [2]. Movement that maximizes neuroplasticity, referred to as activity-dependent neuroplasticity, is repetitive, challenging, and salient to the individual [2]. Numerous rehabilitation interventions, such as body weight-supported treadmill training and upper limb robotic training, have incorporated these characteristics into their design and delivery with the aim of promoting activity-dependent neuroplasticity to improve movement and function.

The emphasis on neurological recovery during SCI rehabilitation led to the development of activity-based therapies (ABT) [3]. ABT target activation of the neuromuscular system affected by the injury, with the goal of retraining the nervous system to recover a specific motor task [3, 4]. Neuromuscular activation is provided during a functional movement since task-specific training is known to contribute, in part, to the recovery of function [2, 3]. Like exercise, which is repetitive and structured physical activity completed with the intent of maintaining or improving fitness [5, 6], many ABT strive for a moderate to vigorous cardiovascular workload, thus promoting fitness and attainment of the recommended levels of exercise for those living with SCI [6]. The potential benefits of ABT among the SCI population are numerous: improvements in mobility, upper limb function, hand function, neurological status, body composition, bowel and bladder function and quality of life, along with a reduction in cardiovascular and metabolic risk factors [3, 7–10].

Defining characteristics of ABT include activation of the neuromuscular system affected by SCI and a high training dosage (i.e. frequency and duration of sessions, number of movement repetitions and exercise intensity). Training at a higher locomotor exercise intensity has been linked to increased peripheral levels of brain-derived neurotrophic factor [11] and greater improvements in locomotor outcomes [12] in individuals with motor incomplete SCI. Most ABT programs involve a minimum of three sessions per week, with each session lasting 90 min or longer [8, 13]. These high training dosages are assumed to result in greater improvements in motor function. Such a dose-response relationship has been demonstrated in ABT for stroke rehabilitation [14]. Among the SCI population, little is known about the training dosages achieved in inpatient and outpatient rehabilitation and there is a paucity of ABT research in the community [15]. The number of movement repetitions and cardiovascular workload achieved during inpatient physical and occupational therapy sessions at the two largest SCI rehabilitation hospitals in Canada were recently studied [16, 17]. The number of upper and lower limb movement repetitions per session, expressed as median (range), were low. For example, inpatients with tetraplegia completed 31 (0–127) upper limb repetitions per occupational therapy session at admission and 2 (0–34) repetitions per session at discharge [16]. Ambulatory inpatients with SCI took a mere 51 steps (0–76) per physical therapy session at admission and 115 steps per physical therapy session (21–313) at discharge [16]. Further, inpatients with SCI spent 80–90% of their therapy sessions below the cardiovascular training zone [17]. Hence, high training dosages are likely rarely achieved in current Canadian rehabilitation programs.

To stimulate advances in ABT research and care in Canada, an ABT Summit sponsored by the Praxis Spinal Cord Institute was held in 2019 to create a Canadian ABT Strategy for SCI. Individuals with lived experience, frontline clinicians, healthcare administrators, researchers, funders of SCI research and health policy experts participated in the meeting. The summit aimed to synthesize current knowledge about ABT in Canada as well as identify what is needed to advance the field and inform a five-year action plan. The summit objectives were to: (1) Review the evolution of similar work in stroke rehabilitation, which may serve as a model, and identify potential methods for SCI. (2) Understand the current state of ABT activities in Canada across the continuum of care. (3) Identify priorities for ABT research and care in Canada for the next five years. Here we report the processes and outcomes of the summit, which form the Canadian ABT Strategy for SCI that is currently being actioned by the Canadian ABT Community of Practice.

## Methods

A summit meeting lasting 1.5 days occurred in March 2019 in Toronto ON, Canada. A participatory design approach was followed such that the meeting consisted of knowledge-sharing presentations amongst a diverse group of stakeholders, as well as small and large group activities. The meeting was facilitated by the three summit leads (VN, KW, and KEM). Two summit attendees (HJR and NT) were designated note-takers that recorded the content of the summit.

### Summit participants

Thirty-seven individuals attended the summit meeting in person and two individuals participated virtually (six provinces represented). For the full list of summit attendees, including their areas of expertise and affiliations, see Supplementary file 1. Attendees included individuals from the following stakeholder groups: (1) people living with SCI, (2) health care administrators/health policy experts, (3) frontline clinicians in publicly-funded acute and rehabilitation hospitals (i.e. physicians, occupational therapists, physical therapists), and in community-based ABT clinics (e.g. kinesiologists, certified exercise physiologist), (4) innovators (e.g. biomedical engineers, technology innovators, funders of SCI research), and (5) researchers with expertise in the following areas: SCI rehabilitation, behavior change, implementation science, stroke rehabilitation, and economic analyses. All five attendees living with SCI had sustained their injury more than 6 years prior to the summit and used a wheelchair for mobility; one used a power wheelchair and attended with a care partner, while four used manual wheelchairs. One attendee was living with paraplegia and four had tetraplegia. Summit participants were invited by the summit leads, who aimed to recruit approximately 5–10 individuals from each stakeholder group while also ensuring representation from western, central and eastern Canada.

### Summit activities

Summit activities focused on exploring the current state of ABT for SCI in Canada and considered potential priorities for ABT research and care over the next five years. The FAME (feasibility, appropriateness, meaningfulness, effectiveness, economic evidence) Framework [18, 19] was used to organize the structure of presentations and group activities. This framework has been used to aid the implementation of evidence-based interventions and technologies in clinical practice [18] and to guide the development and research of novel interventions and technologies [19]. The FAME Framework suggests elements to evaluate as technologies/interventions are developed and/or implemented. See Table 1 for an explanation of the FAME Framework in the context of ABT, and Table 2 for a linking of summit topics to FAME elements.

**Table 1:** FAME Framework [18, 19] and application to Activity-based Therapies (ABT).

FAME Element	Example Applications to ABT
**F**easibility	- How can ABT be successfully implemented into SCI rehabilitation across the continuum of care (i.e. acute care, inpatient and outpatient rehabilitation, community rehabilitation) given the cultural and physical contexts? - What are the barriers and facilitators to ABT implementation?
**A**ppropriateness	- Does ABT fit the therapeutic scenario and Canadian health care context? - Is ABT appropriate for the acute care, inpatient/outpatient rehabilitation, and community-based settings?
**M**eaningfulness	- Does ABT and its intended outcomes matter to end-users (i.e. persons living with SCI)? - Have the personal experiences, perceptions, values, and beliefs of end-users been considered?
**E**fficacy	- Does ABT achieve the expected health effect(s)? Health effects may include clinical and/or health service outcomes
**E**conomic **E**vidence	- Is implementation of ABT across the continuum of care supported given the costs?

**Table 2:** ABT Summit Meeting Agenda. Initials listed in the middle column refer to the summit attendees who led the knowledge-sharing activity.

Knowledge	Knowledge Sharing Activity	FAME Element(s)
*Day 1 (half day)*		
*Review of Relevant Background Information* - Motivation for, and aims of, summit - Review of the literature on ABT - Technologies for assessment and monitoring	Presentation (KEM, KW, VN, JZ, LB)	Feasibility, Appropriateness
*Overview of Efforts to Increase Training Dosage in Stroke Rehabilitation* - Evaluation of training dosage in stroke rehab - Development of upper and lower limb interventions to increase and measure intensity - Translation of interventions	Presentation (JE, TK)	Feasibility, Appropriateness, Efficacy
*Leveraging Lessons Learned from Stroke Rehabilitation* - Identification of strengths of ABT-related research in stroke that may benefit ABT for SCI/D	Large group discussion	Appropriateness
*Overview of Summit Framework & Mapping Exercise* - Introduction of FAME Framework - Introduction of process for documenting current ABT research and care activities in Canada (i.e. mapping exercise)	Presentation (KEM, KW)	Not applicable
*ABT in Acute Care* - Perspectives of clinicians working in acute care: ○ How do you see ABT fitting into SCI acute care? ○ What are the main barriers to implementation of ABT into acute care?	Moderated panel (JC, HJ, CG) Large group discussion	Appropriateness
*Overview of Training Dosage in SCI Rehab* ○ Evaluation of training dosage in SCI rehab	Presentation (DZ)	Appropriateness
*ABT in the Community* - Perspectives of clinicians and clinic managers offering ABT in the community ○ Briefly describe your community-based clinic. ○ What advice would you give someone planning to set-up a community-based facility that offers ABT to people with SCI?	Moderated panel (JL, SM) Large group discussion	Appropriateness
*Implementing Community-based Physical Activity for SCI/D* - Lessons learned from the development and implementation of physical activity guidelines for SCI	Presentation (KMG)	Feasibility, Appropriateness, Efficacy
*Day 2 (full day)*		
*Recap & Review of Current ABT Activities in Canada* - Highlight attendees’ ABT research activities - Highlight attendees’ ABT care activities	Presentation (DW, KM, CP, CH)	Feasibility, Appropriateness, Efficacy
*ABT Across the Continuum of Care* - Perspectives of people living with SCI ○ What are your experiences with ABT and/or engagement in exercise? ○ What is your vision for ABT for the SCI population in five years?	Moderated panel (AK, TJ, GW, IM) Large group discussion	Meaningfulness
*Charting our Course* - Create a vision for success in ABT research and care - Identify what is feasible in 1–2 years and 3–5 years - Consider how current ABT research and care initiatives can be leveraged to achieve success	Small group activity Large group discussion	Meaningfulness, Feasibility
*Brainstorm solutions* - Role of innovation - Role of behavior change - Role of economic data/analyses - Lessons learned from prior implementation projects in SCI	Presentation (TL, LP, SD, BC, PA, DW) Large group discussion	Feasibility, Appropriateness, Efficacy, Economic Evidence
*Charting our Course: Revisited* - Consider how possible solutions from the brainstorming session can be leveraged to achieve success	Large group discussion	Feasibility, Appropriateness, Efficacy, Economic Evidence
*Establishing Priorities* - Finalize a list of possible priorities for ABT research and care	Large group discussion Vote (individual activity)	Not applicable
*Next Steps and Feedback* - Review accomplishments of summit and outline next steps - Written (i.e. survey) feedback collected	Presentation (KEM, KW, VN) Large group discussion Feedback survey (individual activity)	Not applicable

See the Appendix for the full names of summit attendees.

Summit attendees discussed and identified the strengths of the prior work in stroke rehabilitation that may be applicable to the research on, and implementation of, ABT in the SCI field (summit objective 1). A mapping exercise was completed to document the ABT research and clinical care activities that were currently in progress across Canada (summit objective 2). To further describe the current state of ABT across the continuum of care, moderated panels and presentations were included. For SCI in Canada, the continuum of care includes acute care (i.e. emergency and acute inpatient services) and hospital-based rehabilitation services (i.e. inpatient and outpatient rehabilitation) provided by provincial and territorial governments, as well as for-profit and non-profit community-based services that are typically accessed following discharge from hospital-based rehabilitation. Panels consisting of 2–3 summit members each were formed to discuss ABT in acute care and community settings, as well as the lived experience with ABT and/or engagement in exercise. See Table 2 for panelists and discussion questions. Presentations by summit attendees concerning their expertise, research, and care activities related to ABT also contributed to our understanding of the current state of ABT across the continuum of care in Canada (summit objective 2). These presentations demonstrated how the group’s strengths could be leveraged to advance ABT research and care in Canada.

### Generation of priorities

To begin generating priorities (summit objective 3), summit attendees were divided into five stakeholder groups: individuals with SCI (*n* = 5), innovators (*n* = 7), health care administrators/health policy experts (*n* = 8), researchers (*n* = 7), and frontline clinicians (*n* = 10). As some participants identified with more than one group, the assignment was completed with the aim of maintaining roughly equal numbers and geographical diversity within each group. Each stakeholder group was asked to discuss the following two questions: What would success look like for research? What would success look like for care? Following discussion, groups were asked to use a standardized form to generate a list of priorities for each of research and care, organizing the priorities according to what was feasible to complete in 1–2 years versus 3–5 years. Generation and sharing of priority lists by each stakeholder group ensured that each group’s perspectives were reflected in the large group discussion that followed.

At the end of the summit meeting, each attendee was asked to provide written feedback on the priorities through an anonymous and confidential voting process. Participants voted for the top priority in each of the following categories: research in the next 1–2 years, research in the next 3–5 years, care in the next 1–2 years and care in the next 3–5 years. Participants were also invited to provide additional, open-ended feedback concerning their selection of priorities. After the summit, one of the summit leads (KEM) tallied the votes for each category, with the aim of identifying the four priorities (one per category) with the highest number of votes. She also completed a conventional content analysis [20] on the text returned through the voting process, the list of priorities submitted by each stakeholder group and the notes taken by the two note-takers. The synthesis of these data sources identified additional considerations regarding the priorities.

## Results

### Increasing training dosage in stroke rehabilitation: strengths and applicability to SCI

Summit attendees identified numerous strengths of the prior initiatives in stroke rehabilitation (Table 3). Many strengths were deemed applicable to SCI research and care, and provided points to consider as the priorities for ABT research and care were generated.

**Table 3:** Leveraging lessons learned from stroke rehabilitation.

Strengths of initiatives in stroke	Applicability to SCI
Established operational definitions (e.g. intensity)	Consider definition of ABT across continuum of care (e.g. what is an appropriate definition of ABT in the acute phase?) Consider clear definition of intensity that possesses clinical utility
Established clinically-feasible measurements [33]	Consider available tools (e.g. wearable sensors)
Established dose-response relationship [34]	Dose-response relationship may vary throughout phases of SCI recovery ‘Window of opportunity’ for SCI likely not as definitive as that for stroke
Adopted logical approach (i.e. safety → efficacy → translation)	Efficacy data lacking
Built upon pre-existing guidelines to create widely-used protocols (e.g. GRASP [35])	Programs for stroke could be modified for SCI
Recognized need for individualized approach given heterogeneity of stroke population	SCI also requires individualized approach
Recognized need to incorporate behavior change for healthcare professionals	Need for education concerning potential for improvement amongst all individuals with SCI, regardless of injury severity
Recognized importance of partnerships	SCI research and care fields are small, partnerships important
Recognized system-level trends and worked within existing healthcare system (e.g. considered decreasing lengths of stay and need for rehab in the home/community) [36]	Strategies used to increase intensity in stroke rehab could apply to SCI rehab Need for ABT that does not rely on specialized equipment/technology

### Current state of ABT activities across continuum of care in Canada

**Fig. 1:**
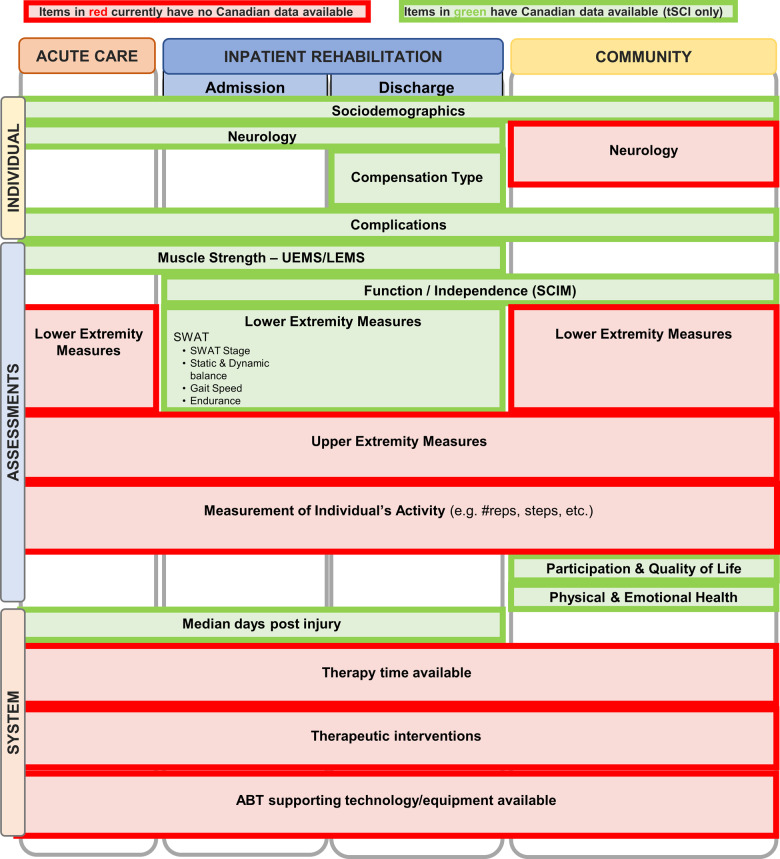
Mapping exercise of activity-based therapies (ABT) across the continuum of care. Horizontally along the top, the continuum of care (i.e. acute care, inpatient rehabilitation, and community) is represented. Vertically, categories include information related to the individual, clinical assessments, and health system factors that may help to inform appropriate and feasible areas for implementation of ABT. Green shading indicates that Canadian data exists for that construct. Red shading indicates that Canadian data does not exist. *tSCI* traumatic spinal cord injury, *UEMS* upper extremity motor score, *LEMS* lower extremity motor score, *SCIM* Spinal Cord Independence Measure, *SWAT* Standing and Walking Assessment Tool.

The mapping exercise identified areas in which Canadian ABT activities and data currently exist and areas that may be lacking activities and data (green and red shading, respectively, Fig. 1). Summit attendees identified numerous single–center initiatives that were not coordinated or implemented at multiple sites. The majority of national-level data have been collected through the Rick Hansen SCI Registry [21], which has been focused on the traumatic SCI population. Data are lacking for individuals with non-traumatic injuries. There is a paucity of known initiatives and data in the acute and community settings, as well as at the health system level. When considering ABT assessments, the Standing and Walking Assessment Tool (SWAT) [22], was recognized as a Canada-wide method for collecting data related to the lower limbs during inpatient rehabilitation. The use of standardized measures for the upper extremity and activity levels were identified as gaps for ABT assessments in Canada.

The members of the acute care panel spoke of numerous barriers to the implementation of ABT in the acute setting: patients’ medical instability and medical complexity (e.g. multi-trauma, intubation), high staff turnover in the acute care environment and limited time for occupational and physical therapy. A high training dosage was not thought to be an appropriate focus for this setting; however, the panelists had several ideas for how ABT could fit within the acute care environment. Specifically, acute care may be an appropriate time to prepare patients, both physically and mentally, for ABT. For example, physical preparation might include maintaining range of motion to facilitate participation in treadmill training at a later stage of recovery. Mental preparation may include educating patients about ABT so that they know what to expect in rehabilitation. The panelists also stressed the importance of messaging that retains hope for patients during this early stage post-SCI.

Members of the community panel shared four pieces of advice for others planning to establish a community-based ABT facility. First, as non-profit charities, they were eligible for grants, donations, and fundraising that enabled them to acquire equipment and staff training. Second, they emphasized the need to focus on patient safety and achieve spotless safety records. Third, they also emphasized the value of the social aspects of community-based ABT facilities; this is an environment where patients obtain peer support and create social connections. Lastly, the community panelists highlighted the importance of establishing supportive relationships, and having open communication, with their local rehabilitation hospitals and other community centers (e.g. YMCA).

### Priority identification

The panel of individuals with SCI shared their vision for ABT in five years. They hope ABT will be viewed as an integral part of rehabilitation after SCI, just like transfer training, for example. If ABT is part of regular clinical practice, it will no longer be viewed as an additional activity that requires extra time, which panelists felt was a barrier to its use. Their vision also includes effective mechanisms for patient education and knowledge translation concerning ABT, and greater access to ABT in the community.

The summit attendees decided against separating priorities into research and care since research and clinical care are integrated entities that involve bidirectional flow of information. The five identified priorities (Table 4), presented in order from shorter-term to longer-term vision, were: (1) Identifying current ABT activities across the continuum of care (16 votes); (2) Creating a network to facilitate dialog across the continuum of care (23 votes); (3) Tracking engagement in ABT activities across the continuum of care (19 votes); (4) Development and implementation of best practice recommendations for ABT (34 votes); and (5) Study of optimal timing, methods, and dose of ABT to promote desired outcomes (18 votes). Eight cross-cutting categories were identified through the conventional content analysis (Table 4). These categories reflect important concepts identified by summit attendees that should be considered and incorporated into activities that will flow from the priorities above.

**Table 4:** Priorities for ABT Research and Care in Canada.

Priorities
**1) Identifying current ABT activities across the continuum of care** - A snapshot of the current state - Characterize technologies, interventions and physical activity programming (that meet ABT criteria) - Share the ABT activities used by sites with all sites
**2) Creating a network to facilitate dialog across the continuum of care** - Create a database of community facilities offering ABT that can be a resource for hospital clinicians and individuals with SCI - Establish linkages between hospitals and community facilities to share knowledge and maintain regular communication
**3) Tracking engagement in ABT activities across the continuum of care** - Identify or develop a standardized tool/method for documentation - Define intensity - Standardize measures for data collection across clinical sites, including community - Use data to answer questions about outcomes of ABT, include qualitative research to gauge meaningfulness
**4) Development and implementation of best practice recommendations for ABT** - Establish common terminology - Develop criteria/metrics for ABT delivery - Support clinical implementation of best practice recommendations
**5) Study of optimal timing, methods and dose of ABT to promote desired outcomes** - Summarize research evidence that already exists - Decision support concerning individualized approach - Build neurophysiological evidence
**Cross-cutting Categories**
a) Include the upper limb, not only the lower limb b) Include behavior change strategies - At multiple levels as appropriate: individual (clinicians and persons living with SCI), institutional (health care and academic), community, policy - Frequent mention of need to influence the training of current and future health care workers - iKT for institutional, community, policy levels - Involve peers c) Include economic analyses d) Produce rigorous and valid research - Research that has the potential to impact policy change e) Incorporate reliable technology for motivation, intensity, engagement, monitoring f) Link data g) Create innovative collaborations, connect expertise h) When appropriate, build upon methods/protocols used in stroke research and practice

## Discussion

The ABT Summit participants were tasked with identifying priorities for ABT research and clinical care, with a long-term vision of increasing the access to, and quality of, ABT for Canadians living with SCI. Five priorities were generated that recognized the need for: cooperation and communication across the continuum of care, increased knowledge of current ABT activities, standardized methods to track participation and outcomes in ABT, the generation of new knowledge concerning dose and timing of ABT, and the development of clinical recommendations for ABT. To achieve these goals, it was recognized that behavioral change strategies, economic analyses, technology innovations, collaborations, and data linkages would be required.

Following the 2019 summit meeting, working groups were formed to address the first three priorities for ABT research and clinical care (Table 4). In the fall of 2020, the summit attendees and additional working group members formed the Canadian ABT Community of Practice, and established a steering committee and terms of reference to coordinate efforts to address the summit priorities. Identifying current ABT activities and creating a network to facilitate dialog across the continuum of care may be achieved on a shorter timescale (1–2 years) than the latter three priorities. The actionable items of the working groups included a combination of research and knowledge translation. Knowledge sharing activities, such as the Canadian ABT Expo and Spinal Moves podcast [23], were created to increase awareness of ABT amongst all stakeholders, including family members and caregivers. Research activities included: (1) a scoping review to identify the characteristics of current ABT activities internationally [24]; (2) an inventory of the ABT-related technology available at hospitals and community clinics that work with the Canadian SCI population; and (3) exploratory qualitative studies that aimed to understand if, and how, ABT is incorporated into the rehabilitation of Canadians living with SCI [25, 26].

The summit highlighted a potential role for ABT across the continuum of care, including in acute care. As outlined by the acute care panelists, acute care clinicians have the potential to play an important preparatory role for ABT. Exploring if and how ABT is operationalized in the acute care environment would be beneficial, along with consideration of methods of ABT delivery that may be particularly appropriate for acute care, such as motor imagery. There is preliminary evidence that motor imagery can normalize cortical recruitment and decrease movement variability in individuals with cervical SCI [27]. Further, individuals with SCI have described the importance of mental effort in their community- and home-based ABT programs [25].

The latter three priorities for ABT research and clinical care (Table 4) are broader in scope and sequential in nature. The development of tools to track engagement in ABT would benefit research concerning the optimal timing, methods, and dose of ABT, and this research would benefit the development of best practice recommendations. As a result, we expect these latter three priorities will be realized over the next 3–5 years. The summit attendees acknowledged that the development and implementation of best practice recommendations would require collaboration with academic institutions and health professional credentialing bodies, and that connections with these bodies should be an immediate priority. Numerous summit attendees are involved in the education of Canadian physical therapists or physicians, have leadership roles within their professional associations, and/or have prior experience with the development of indicators of high-quality rehabilitation for Canadians with SCI [28]. These experiences are expected to facilitate the relationship building that will be crucial for the meaningful dissemination of ABT best practice recommendations.

There were several strengths of the process used to generate the priorities for ABT research and care in Canada. First, best practices for stakeholder engagement were followed [29, 30]. For example, there was a balanced representation of numerous stakeholder groups, which allowed the inclusion of a variety of perspectives. The summit organizers ensured participants understood their role in the summit through individual dialog prior to the meeting. Through the small and large group discussions, connections were established within and between stakeholder groups. The choice to hold an in-person meeting, rather than virtual, may have facilitated the development of connections with face-to-face interactions enabling greater spontaneous interactions between participants. These connections were further supported through sustained stakeholder engagement post-summit, which included the formation of working groups and webinars on ABT topics by summit attendees. A second strength of the process used to generate the priorities was the inclusion of individual, anonymous voting following consensus building activities. This voting process ensured the opinions of all participants were included.

Upon reflection, there was at least one stakeholder group missing from the summit, which is a limitation of our process. Family members of people living with SCI were not targeted for inclusion in the summit, yet their support is likely important for ABT participation. The social support and encouragement of friends and family are known to influence whether physical rehabilitation is perceived as having a positive or negative impact by individuals living with SCI [31]. When considering ABT specifically, a previous study found that several participants of an intensive locomotor training program reported they could not have participated in the program had it not been for their spouse and family members [32]. The family was reported to provide motivation, assist with transportation to and from the rehabilitation facility, and to assist with self-care tasks, such as bowel care, while at the facility [32]. The Canadian ABT Community of Practice has and will continue to include family members and informal caregivers in subsequent initiatives. Another potential limitation to note is the limited range of impairments and chronic injury status of the attendees with SCI, in part due to the small number of attendees with SCI. As a result, the collective range of experiences with inpatient and outpatient rehabilitation and/or ABT may have been limited. In recognition of this limitation, a working group was created to collect the perspectives of a more heterogeneous group of individuals with SCI [25]. Lastly, the recommendations from the summit were developed by stakeholders to address the concerns facing a health care system that is predominantly funded, delivered, and regulated by the provinces and territories of Canada. As a result, some of the priorities identified at a national level, such as improved communication throughout the continuum of care and knowledge of current ABT activities, are a response to the fractured nature of the Canadian health care system. Health care systems in different jurisdictions may identify different priorities. Similarly, the structure of a publicly funded Canadian health care system, will introduce unique facilitators and barriers in the implementation of ABT. For example, a single health care payer may facilitate the integration of ABT province-wide where there are funding commitments in place. However, the necessary funding required to sufficiently provide these interventions to those in need at a provincial level discourage investment in ABT.

In conclusion, the priorities for ABT research and care for Canadians living with SCI were identified through a stakeholder-engaged summit meeting. These priorities will guide the activities of the Canadian ABT Community of Practice over the next five years to increase the access to, and quality of, ABT for Canadians living with SCI.

## Supplementary information

Appendix

## Data Availability

The datasets generated and/or analyzed during the current study are available from the corresponding author on reasonable request.
