# Supplementary material for: Development of priorities for a Canadian strategy to advance activity-based therapies after spinal cord injury
Source: Spinal Cord. 2021 Jun 7;59(8):874–84. doi: 10.1038/s41393-021-00644-2 (PMC8338546; doi:10.1038/s41393-021-00644-2)
Supplement: Supplementary file 1 — Appendix [file 41393_2021_644_MOESM1_ESM.docx]

**Supplementary file 1**: ABT Summit Meeting Attendees

The Canadian ABT Summit Meeting attendees, presented in alphabetical order by last name. Name (left column), area(s) of expertise (middle column) and affiliation(s) (right column) are reported. ABT = activity-based therapies; HINRI = Healthcare Institute for NeuroRecovery and Innovation; SCI = spinal cord injury; UHN = University Health Network.

| **Participant** | **Area(s) of expertise** | **Affiliation(s)** |
| --- | --- | --- |
| Peter Athanasopoulous | Peer support, public policy, government relations | SCI Ontario, Ontario SCI Solutions Alliance, Toronto, ON |
| Dr. Laurent Bouyer, PhD | Motor control, motor learning | Laval University, Center for Interdisciplinary Research in Rehabilitation and Social Integration, Quebec City, QC |
| Dr. Brian Chan, PhD | Economic analyses, decision modelling, cost-of-illness studies | UHN-KITE, Toronto, ON |
| John Cobb, OT | Occupational therapy in rehabilitation and community phases | Vancouver General Hospital, Vancouver, BC |
| Lorie Crouse, OT | Occupational therapy, implementation of best practice initiatives | Glenrose Rehabilitation Hospital, Edmonton, AB |
| Dr. Sarah Donkers, PT, PhD | Neurological physical therapy, translational and behavioural neuroscience | School of Rehabilitation Science, University of Saskatchewan, Saskatoon, SK |
| Gwen Dziwenko, OT | Occupational therapy, knowledge mobilization with new technology | Glenrose Rehabilitation Hospital, Edmonton, AB |
| Dr. Janice Eng, PhD | Physical and occupational therapy, arm and hand function, fitness and mobility | University of British Columbia, GF Strong Rehab Centre, Vancouver, BC |
| Heather Flett, PT, MSc | Physical therapy, clinical research integration, best practices in SCI rehab | UHN – Lyndhurst Centre, Ontario Solutions Alliance, Toronto, ON |
| Dr. Chris Grant, MD | Physiatry, trauma and critical care rehabilitation | Foothills Medical Centre, University of Calgary, Calgary, AB |
| Kristina Guy, PT, MSc | Physical therapy in SCI rehab | UHN – Lyndhurst Centre, Toronto, ON |
| Dr. Chester Ho, MD | Health service delivery, management and rehabilitation of complications | University of Alberta |
| Hilary Jebson, PT | Physical therapy in SCI rehab | Vancouver General Hospital, Vancouver, BC |
| Tara Jeji | Research and implementation for SCI program | Ontario Neurotrauma Foundation, Toronto, ON |
| Hope Jervis Rademeyer, PT, PhD(c) | Neurotechnology for people with SCI, physical therapy | University of Toronto, Toronto, ON |
| Anita Kaiser, PhD(c) | ABT, lived experience | Canadian Spinal Research Organization, UHN-KITE, Toronto, ON |
| Dr. Tara Klassen, PT, PhD | Effects of exercise on stroke recovery, physical therapy | University of British Columbia, GF Strong Rehabilitation Centre, Vancouver, BC |
| Marie-Therese Laramee, PT | Physical therapy, knowledge mobilization | Centre-Sud-de-l’île-de-Montréal, Montréal, QC |
| Dr. Jean-François Lemay, PhD | Postural control, neurological physical therapy | Université de Montréal, Centre-Sud-de-l’île-de-Montréal, Montréal, QC |
| Dr. Jennifer Leo, PhD | Physical activity, community-based services, inclusive services | University of Alberta, Edmonton, AB |
| Timothy Luk, MSc | Emerging health technologies, digital health solutions | MaRS Discovery District, Toronto, ON |
| Sandi Marshal, BKin | Kinesiology, ABT | First Steps Wellness Centre, Regina, SK |
| Stephanie Marrocco |  | Parkwood Institute, London, ON |
| Dr. Kathleen Martin Ginis, PhD | Physical activity behaviour change, psychosocial consequences of physical activity participation | University of British Columbia, Kelowna, BC |
| Dr. Kei Masani, PhD | Neuromechanics, assessments and therapeutic tools, functional electrical stimulation | UHN-KITE, University of Toronto, Toronto, ON |
| Shane McCullum, PT | Mobility improvements, functional electrical stimulation, physical therapy | Stan Cassidy Centre for Rehabilitation, Fredericton, NB |
| Ignacio Montoya | Advocacy, restoration of function and independence after SCI | HINRI Labs, Atlanta, GA |
| Dr. Kristin Musselman, PT, PhD | Neurological physical therapy, locomotor training, functional electrical stimulation | UHN-KITE, University of Toronto, Toronto, ON |
| Dr. Vanessa Noonan, PT, PhD | Processes and outcomes of SCI rehabilitation | Praxis Spinal Cord Institute, Vancouver BC |
| Dr. Colleen O’Connell, MD | Treatments and applied technologies for mobility, impairment and function | Dalhousie University, Halifax, NS, University of New Brunswick, Fredericton, NB |
| Louise Pichette, MMI | Critical gaps in clinical and outcome measures | MaRS Discovery District, Toronto, ON |
| Christine Poirier, PT | Neurological physical therapy, knowledge translation | GF Strong Rehabilitation Centre, Vancouver, BC |
| Isabelle Robidoux, PT | Clinical coordination, physical therapy | Centre-Sud-de-l’île-de-Montréal, Montréal, QC |
| Dr. Nancy Thorogood, PhD | Translational research, neuro-restorative therapies | Praxis Spinal Cord Institute, Vancouver, BC |
| Kristen Walden, PT | Research and best practice initiatives, physical therapy | Praxis Spinal Cord Institute, Vancouver, BC |
| Dr. Dalton Wolfe, PhD | Knowledge mobilization and best practice implementation, physical activity and ABT | Parkwood Institute, London, ON |
| George Woodworth | Peer mentorship, lived experience | Stan Cassidy Center, Fredericton, NB |
| Dr. José Zariffa, PhD, P.Eng. | Neuroprosthetics and technology for upper limb neurorehabilitation | UHN-KITE, University of Toronto, Toronto, ON |
| Dr. Dominik Zbogar, PhD | Physical activity during inpatient SCI | Spinal Cord Injury Rehabilitation Evidence, Vancouver, BC |
